# ASSOCIATION BETWEEN LEVEL OF PAIN AND DEPRESSION AMONG CHRONICALLY ILL OLDER ADULTS IN RURAL ALABAMA

**DOI:** 10.1093/geroni/igz038.975

**Published:** 2019-11-08

**Authors:** Hyunjin Noh, Anne Halli-Tierney, Lewis Lee, Temilade Aladeokin

**Affiliations:** 1 The University of Alabama School of Social Work, Tuscaloosa, Alabama, United States; 2 The University of Alabama College of Community Health Sciences, Tuscaloosa, Alabama, United States

## Abstract

Pain and depression, two of the common symptoms among chronically ill older adults, have been found to be related in various populations; however, further knowledge is needed about their relationships and moderating factors among community-dwelling, chronically ill older adults, particularly in lower-income, rural areas with limited healthcare resources. Therefore, this study aimed to examine the association between pain level and depression among chronically ill older adults in rural areas. A total of 100 residents of a rural county in West Alabama, who are 55+ and have chronic illnesses and pain, were recruited from four community senior centers and were interviewed using a structured questionnaire. Pain levels were assessed by the Philadelphia Geriatric Center (PGC) Pain Scale, and depression by an abbreviated version of the Center for Epidemiologic Studies Depression Scale (CES-D). Bivariate correlation and multivariate analysis were conducted. The correlation between pain and depression was significantly positive (r = .35, p < .001). The results of the model indicated that pain scores and other control variables explained approximately 18 percent of the variance in depression. The multivariate analysis results confirmed that those who had higher pain scores were significantly likely to have increased depression scores (b = 4.97, SE = 1.52, p < .01). Education marginally significantly moderated the relationship between pain and depression (p = .059). The previously reported positive pain-depression relationship exists among chronically ill older adults in rural areas, calling for tailored interventions to reduce their pain and its impact on depression.

